# Supplementary figures and images for: Multi-omic signatures of host response associated with presence, type, and outcome of enterococcal bacteremia
Source: mSystems. 2025 Jan 21;10(2):e01471-24. doi: 10.1128/msystems.01471-24 (PMC11834471; doi:10.1128/msystems.01471-24)

*E. faecium*  
*E. faecalis*

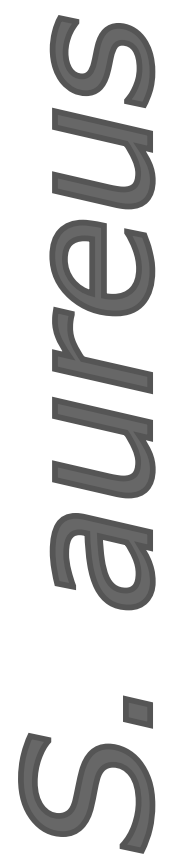

# E. faecium

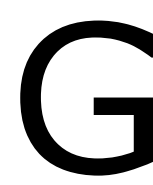

# S. aureus

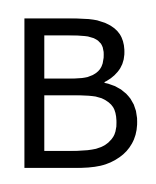

## D

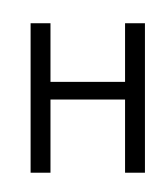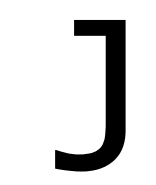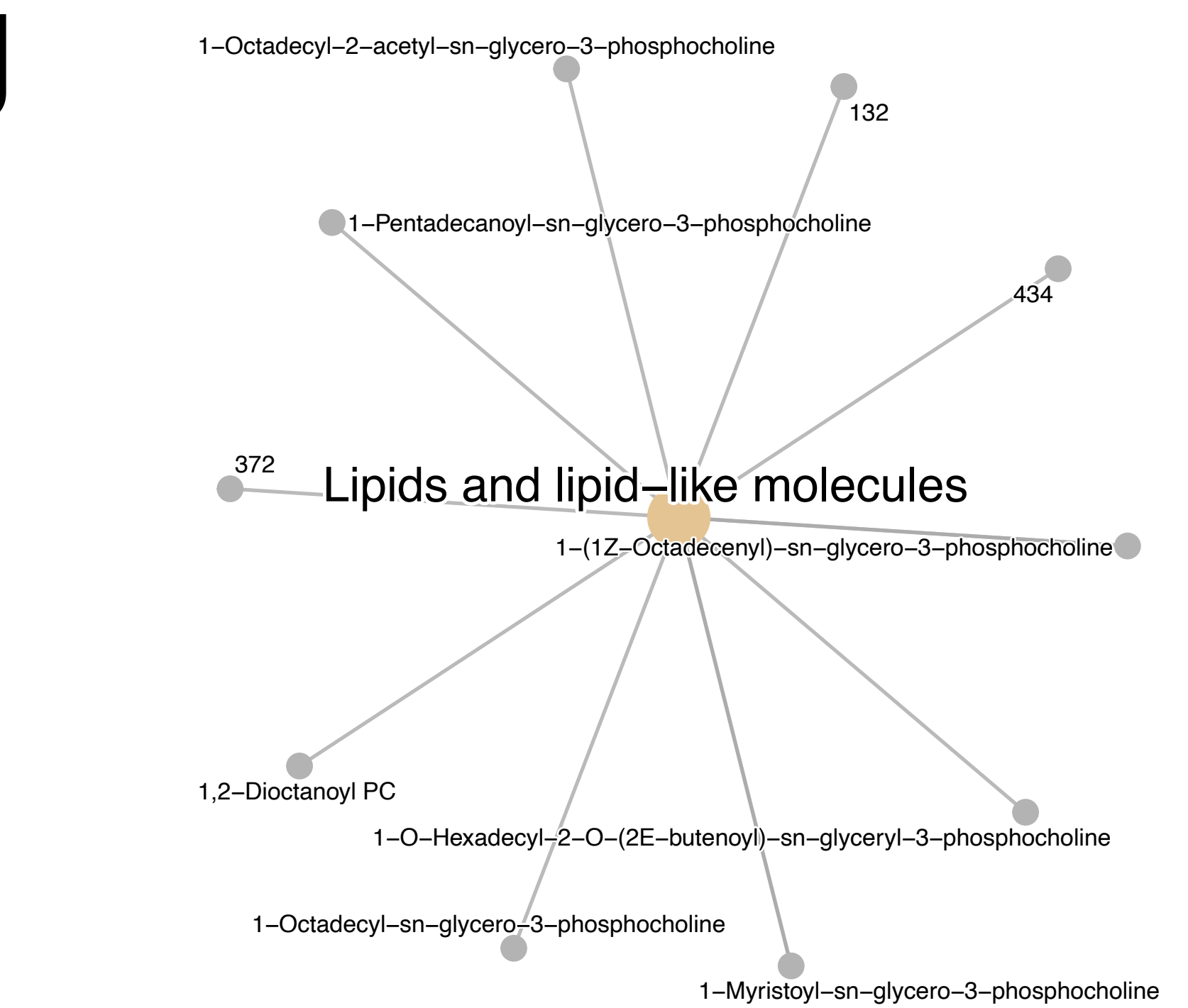

Supplement: Figure S2 — Functional networks of significantly different features. [file msystems.01471-24-s0001.pdf]
