# Supplementary material for: Multi-omic signatures of host response associated with presence, type, and outcome of enterococcal bacteremia
Source: mSystems. 2025 Jan 21;10(2):e01471-24. doi: 10.1128/msystems.01471-24 (PMC11834471; doi:10.1128/msystems.01471-24)
Supplement: Supplemental Material — Supplemental figures and captions for supplemental tables. [file msystems.01471-24-s0002.docx]

Charlie Bayne^1,2,3^, Dominic McGrosso^1,2,3^, Concepcion Sanchez^1,2,3^, Leigh-Ana Rossitto^1,2,3^, Maxwell Patterson^2^, Carlos Gonzalez^2,3^, Courtney Baus^4^, Cecilia Volk^5^, Haoqi Nina Zhao^3^, Pieter Dorrestein^2,3,6,8^, Victor Nizet^2,3,6^, George Sakoulas^6,7^, David J. Gonzalez^2,3,8, #^, Warren Rose^5, #^

Institutions:

^1^Biomedical Sciences Graduate Program, UC San Diego, La Jolla, California 92093, USA.

^2^Department of Pharmacology, University of California San Diego, La Jolla, California 92093, USA

^3^Skaggs School of Pharmacy and Pharmaceutical Sciences, University of California San Diego, La Jolla, California, USA

^4^Department of Pharmacy, UW Health, Madison WI 53792, USA

^5^School of Pharmacy, University of Wisconsin-Madison, Madison, WI 53705, USA

^6^Department of Pediatrics, UC San Diego, La Jolla, California 92093, USA

^7^Sharp Rees Stealy Medical Group, San Diego, CA

^8^Center for Microbiome Innovation, University of California at San Diego, La Jolla, California 92093USA

# co-corresponding authors

Supplementary Material

**Figure S1.** **Overview of Analysis Workflow.**

1. Proteomics analysis workflow.
2. Metabolomics analysis workflow.

*See attached S2.pdf file*

**Figure S2. Functional Networks of Significantly Different Features.**

Networks of features belonging to significantly enriched GO terms (proteomics) or molecular class (metabolomics) (p.adj value <= 0.05 relative to the background universe of all features detected in the study).

1. Increased in EcB compared to healthy volunteers.
2. Increased in healthy volunteers compared to EcB.
3. Increased in *E. faecalis* bacteremia compared to healthy volunteers.
4. Increased in healthy volunteers compared to *E. faecalis* bacteremia*.*
5. Increased in *E. faecium* bacteremia compared to healthy volunteers.
6. Increased in healthy volunteers compared to *E. faecium* bacteremia.
7. Increased in *S. aureus* bacteremia compared to healthy volunteers.
8. Increased in healthy volunteers compared to *S. aureus* bacteremia.
9. Increased in *S. aureus* bacteremia relative to healthy volunteers.
10. Increased in healthy volunteers relative to *S. aureus* bacteremia.

**Figure S3. Evaluation of Clinically Used Biomarkers**

1. ROC curves derived from logistic regression modeling of CRP and SAA1 wherein healthy status was coded as success (1) and EcB status was coded as failure (0).
2. Violin plots showing CRP and SAA1 abundances detected among EcB patients by causative organism and healthy volunteers. Statistics show results of t tests adjusted for multiple comparisons.

**Figure S4. Top 10 Biomarkers for Distinguishing Healthy from Infected**

Comparisons of abundances of the top 10 features identified using EFS with violin plots showing differences in abundance and ROC curves produced by logistic regression wherein healthy status was coded as success (1) and EcB status was coded as failure (0). Violin plot statistics indicate results of t tests adjusted for multiple comparisons.

1. Proteomics
2. Metabolomics

**Figure S5. Top 10 Biomarkers for Distinguishing Faecium from Faecalis**

Comparisons of abundances of the top 10 features identified using EFS with violin plots showing differences in abundance and ROC curves produced by logistic regression wherein *E. faecalis* status was coded as success (1) and *E. faecium* status was coded as failure (0). Violin plot statistics indicate results of t tests adjusted for multiple comparisons.

1. Proteomics
2. Metabolomics

**Figure S6. Top 10 Biomarkers for Distinguishing Mortality from Survival**

Comparisons of abundances of the top 10 features identified using EFS with violin plots showing differences in abundance and ROC curves produced by logistic regression wherein survival status was coded as success (1) and mortality status was coded as failure (0). Violin plot statistics indicate results of t tests adjusted for multiple comparisons.

1. Proteomics
2. Metabolomics

**Figure S7. Assessment of Top 10 Biomarkers for Potentially Confounding Variables**

P-values resulting from testing for significant associations of top identified features with clinical metadata. Categorical metadata associations were determined using Mann-Whitney U test /Wilcoxon rank-sum test (when only two categories were present) or Kruskall-Wallis test (when more than two categories were present). Continuous metadata associations were determined using Pearson correlation.

1. *E. faecalis* vs *E. faecium* top proteomic features.
2. *E. faecalis* vs *E. faecium* top metabolomic features
3. Mortality vs survival top proteomic features
4. Mortality vs survival top metabolomic features


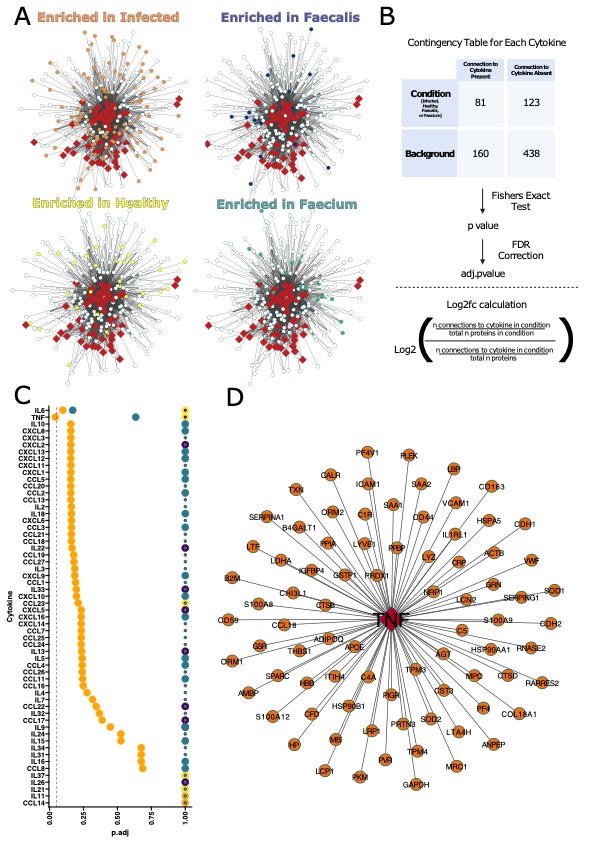


**Figure S8. Cytokine Inference from Significantly Increased Proteins.**

1. Network of proteins and interactions with a panel of cytokines colored by whether they were significantly enriched in EcB infection (compared to healthy volunteers- orange), *E. faecalis* (compared to healthy volunteers- navy), *E. faecium* (compared to healthy volunteers- teal), or healthy volunteers (compared to EcB infection- yellow).
2. Diagram of the logic and statistics used to infer cytokine abundance.
3. P-values and direction of change of cytokines included in cytokine inference screening panel. Size of dots represent direction of change with large being increased, and small being decreased. Colors represent the sample type as described in (A).
4. Network highlighting associations of significantly increased proteins observed in EcB infection and expected interactions with TNF alpha.


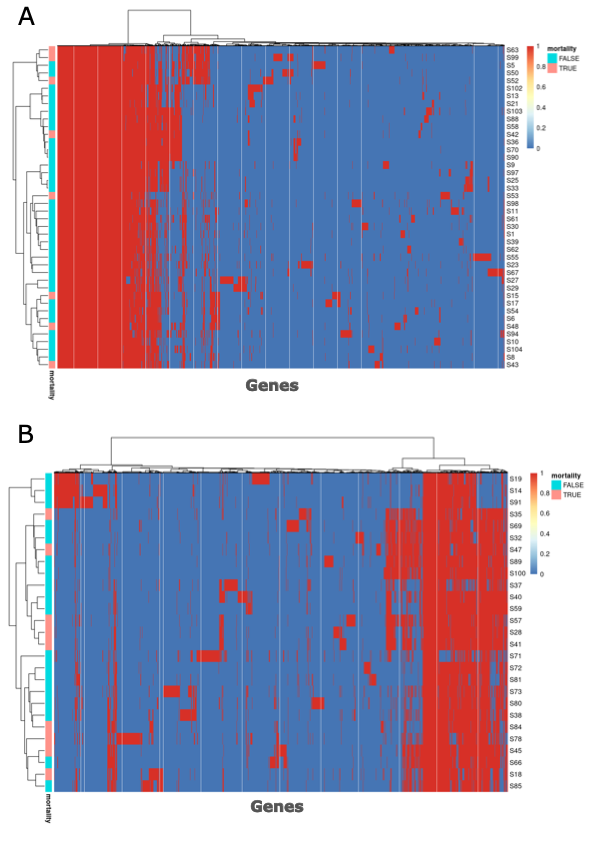


**Figure S9. Clustering of Clinical Isolates of Enterococcus by Gene Content.**

Unsupervised clustering of *de novo* assemblies of strains isolated from EcB patients in this study, as conducted using the binary distance metric and complete agglomeration method. X axis indicates genes in pan genome, color indicates presence (red) or absence (blue) of gene. Colored bars on the Y axis represent whether the patient succumbed to mortality (red) or survived (blue)

1. *E. faecalis* isolates
2. *E. faecium* isolates

**Figure S10. Assessment of Confounding Variable Effects**

1. Differences in immunoglobulin abundances between *E. faecalis* and *E. faecium* bacteremia when filtered to only include patients that had not gotten any organ transplant. Statistics show the results of t tests adjusted for multiple comparisons.
2. Differences in biomarkers for distinguishing *E. faecalis* and *E. faecium* identified to be significantly associated with smoking when filtered to only include nonsmoking patients. Statistics show the results of t tests.
3. Differences in protein biomarkers for distinguishing *E. faecalis* and *E. faecium* identified to be significantly associated with transplant type when filtered to only include patients who had not gotten any organ transplant. Statistics show the results of t tests.
4. Differences in metabolite biomarkers for distinguishing *E. faecalis* and *E. faecium* identified to be significantly associated with transplant type when filtered to only include patients who had not gotten any organ transplant. Statistics show the results of t tests.


**Figure S11. Machine Learning Analysis to Predict Infection, EcB type, and Mortality using Proteomics and Metabolomics Data.**

Lasso regression was conducted using annotated metabolite features from GNPS and proteomic data as predictors. ROC curves illustrate model performance on an unseen test set, accompanied by model coefficients that indicate the most influential predictors for each model:

**(**A) Prediction of infection status

(B) Prediction of EcB type

(C) Prediction of mortality

**Figure S12. Machine Learning Analysis to Predict EcB Type Using Clinical Metadata.**

Lasso regression was performed using fields from the clinical metadata as predictors. ROC curves illustrating model performance on the unseen test set, alongside model coefficients indicating important predictors, are shown for:

(A) Prediction of EcB type using full clinical metadata.

(B) Prediction of EcB type with features unavailable at admission removed.

**Figure S13. Machine Learning Analysis to Predict Mortality Type Using Clinical Metadata.**

Lasso regression was performed using fields from the clinical metadata as predictors. The figure includes ROC curves illustrating model classification on the unseen test set, along with model coefficients indicating key predictors, for:

(A) Prediction of mortality using full clinical metadata.

(B) Prediction of mortality with features unavailable at admission removed.

*See attached ST1_proteomic_stats.xlsx file*

**Table S1.** Protein level comparisons between groups. Statistics were performed on the output of Fragpipe using the MSStatsTMT R package. Three comparisons are reported, healthy volunteers vs EcB infected, E. faecalis vs E. faecium, and mortality vs survival.

*See attached ST2_metabolomic_stats.xlsx file*

**Table S2.** Metabolite comparisons between groups. Statistics were performed on normalized metabolomics data for each feature using t tests as implemented in the rstatix R package. FDR correction was then applied to account for multiple comparisons. Three comparisons are reported, healthy volunteers vs EcB infected, E. faecalis vs E. faecium, and mortality vs survival.
